# Effect of Extruder Screw Speed, Temperature, and Enzyme Levels on Sugar Recovery from Different Biomasses

**DOI:** 10.5402/2013/942810

**Published:** 2012-05-29

**Authors:** Chinnadurai Karunanithy, Kasiviswanathan Muthukumarappan, William R. Gibbons

**Affiliations:** Department of Agricultural Biosystems Engineering, South Dakota State University, 1400 North Campus Drive, Brookings, SD 57007, USA

## Abstract

Biofuels from biomass have the potential to reduce the dependency on fossil fuels. An efficient pretreatment method is required to accomplish the target of the Energy Act 2005. Extrusion could be a viable continuous pretreatment method to be explored. The objectives of the current study were to investigate the influence of screw speed and barrel temperature on sugar recovery from the selected warm season grasses and to select a suitable enzyme combination and dose for enzymatic hydrolysis. The ground, moisture-balanced biomasses were pretreated using a single screw extruder at various screw speeds (100, 150, and 200 rpm) and barrel temperatures (50, 75, 100, 150, and 200°C). Cellulase or multienzyme with *β*-glucosidase was varied from 1 : 1 to 1 : 4 during enzymatic hydrolysis to accomplish the second objective. Screw speed, barrel temperature, and their interaction had a significant influence on sugar recovery from the selected biomasses. A maximum of 28.2, 66.2, and 49.2% of combined sugar recoverywasachieved for switchgrass, big bluestem, prairie cord grass when pretreated at a screw speed of 200, 200, and 150 rpm and at a barrel temperature of 75, 150, and 100°C, respectively, using cellulase and *β*-glucosidase at a ratio of 1 :  4. Extrusion pretreatment of these biomasses used only 28–37% of the rated extruder power.

## 1. Introduction

Renewable energy from biomass has the potential to reduce dependency on fossil fuels, in addition to combat the environmental issues. The Energy Policy Act of 2005 mandates blending of 7.5 billion gallons of alternative (biofuel) fuels by 2012 [1]. The biofuels such as ethanol and biodiesel available in the market are predominantly produced from corn, sugar cane, and soybean oil [2]. It has been reported that biomass and bioenergy provides only about 4% of the total primary energy used in the US [3]. Lignocellulosic materials are the most abundant renewable resources on earth [4]. According to Kadam and McMillan [5], about 80–100 dry tons of corn stover/year can be utilized for ethanol production. Corn stover was the most researched biomass and it alone might not be able to fulfill the fuel requirement of the US. Warm season grasses such as switchgrass, big bluestem, and prairie cord grass are grown in most part of the nation. In general, the warm season grasses have higher sugar content than cool season grasses [6]. Several studies have indicated that these warm season grasses have greater potential as feedstock to produce biofuels [6–8]. The sugars in lignocellulosic materials mostly exist as polysaccharides such as cellulose and hemicellulose, which are not readily available for hydrolytic enzymes. Pretreatment is an inevitable first step in the conversion of biomass to biofuels and the most expensive step too.

The focus of pretreatment research is to develop processes that would reduce the bioconversion time, lower the cellulase enzyme usage, and increase ethanol yields [9]. Several pretreatment methods such as dilute acid, alkali, organic solvents, wet oxidation, ammonia fiber explosion, hydrothermal (water, steam), milling, and irradiation have been investigated with varying degrees of success for the past three decades. No perfect pretreatment method has been established to produce biofuels from biomass on commercial scale [10]. Extrusion might be a viable continuous pretreatment method, which has many advantages such as high shear, rapid mixing, short residence time, moderate barrel temperature, no furfural and HMF formation, no washing and conditioning, and adaptability to process modification. A few extrusion pretreatments showed a significant improvement on sugar recovery from different biomasses such as corn stover [11, 12], miscanthus [13], switchgrass, prairie cord grass, big bluestem [14, 15], and Douglas fir [16] through enzymatic hydrolysis.

Enzyme loading is an expensive input in biomass conversion [17–19]; hence, enzyme usage should be as low as possible. Typically enzymatic hydrolysis of cellulose is carried out using cellulase—a complex system consists of three enzymes, namely, endoglucanase, exoglucanase, and cellobiase that act synergistically. The cellobiase (*β*-glucosidase) supplementation with cellulase enzyme was necessary to eliminate the inhibition effect of cellobiose [20, 21]. The removal of cellobiose by *β*-glucosidase results in the absence of product inhibition [22, 23], thus, hydrolysis can be achieved at reduced enzyme levels. Varga et al. [24] reported a glucose conversion of 78.5–81.2% with complementation of *β*-glucosidase whereas it was only 57.8% without complementation of *β*-glucosidase when corn stover was pretreated using wet oxidation. A wide range of enzyme dosage and hydrolysis conditions has been reported in literature for different biomasses depending on pretreatment. Due to variation in enzyme activity and its dose, making meaningful comparison between pretreatment methods is very difficult. Hence, there is a need to determine the suitable enzymes and their doses for economic reasons.

The objective of the current study is to address the two most expensive steps such as pretreatment and enzymatic hydrolysis in the biomass conversion process. The first objective is to evaluate the effect of screw speed and barrel temperature on sugar recovery from warm season grasses such as switchgrass, big bluestem, and prairie cord grass. The second objective is to select a suitable enzyme combination and their ratio for the enzymatic hydrolysis of pretreated switchgrass, big bluestem, and prairie cord grass.

## 2. Materials and Methods

### 2.1. Biomass Preparation

Switchgrass, big bluestem, and prairie cord grass are three major warm season grasses among the big four native grasses (other: indiangrass) in South Dakota with potential as biomass feedstocks [25]. Switchgrass, big bluestem, and prairie cord grass obtained from a local farm were ground in a hammer mill (Speedy Jr, Winona Attrition Mill Co, MN) using 4 mm sieve for further pretreatment. The moisture content was determined as described by Sluiter et al. [26]. The moisture content of ground biomass was adjusted to 21% (w.b) based on preliminary study. Compositional analysis of biomass was carried out as outlined by Sluiter et al. [27, 28].

### 2.2. Extrusion Pretreatment

Extrusion was performed in a single screw extruder (Brabender Plasti-Corder Extruder Model PL2000, Hackensack, NJ), which had a compression ratio of 3 : 1, barrel length to screw diameter ratio (*l*/*d*) of 20 : 1. In order to have a smooth biomass (plug) flow into the die section, the screw discharge end was fitted with conical metal piece. The single screw extruder was fitted to a 7.5 hp motor, which had a provision to adjust the screw speed from 0 to 210 rpm. The screw speed of the extruder was maintained at 100, 150, and 200 rpm during the extrusion of biomass samples. The extruder barrel had provisions to control the temperature of the feed and transition zone in both barrel and die section. The transition zone and die section temperature of barrel was maintained between 50, 75, 100, 150, and 200°C for different screw speeds depending upon the biomass. The temperature inside the barrel and the speed of the screw were controlled by a computer; and feeding to the extruder was done manually. Compressed air was supplied as a cooling agent along the barrel length. About 500 g of prairie cord grass was extruded under each pretreatment condition, divided into two batches accounting for variations due to extruder operation, and considered replicates. The mean residence time varied between 30 and 90 sec depending upon the screw speed. The power consumption for extrusion pretreatment of different feedstocks was measured clamp meter (Amprobe ACD-4, Everett, WA).

### 2.3. Enzymatic Hydrolysis

The enzymatic hydrolyses were conducted in hungate glass tube (Bellco glass, Inc, NJ, USA) with 0.3 g dry weight of pretreated biomass in a solution of citrate buffer (0.05 M, pH 4.8) and sodium azide (0.02 gL^−1^) to maintain constant pH and to inhibit microbial contamination, respectively. In order to select an enzyme combination and ratio, multienzyme (activity 100 FBGg^−1^), cellulase (activity 70 FPUg^−1^) with *β*-glucosidase (activity 250 CBUg^−1^) in the ratio of 1 : 1 and 1 : 4 was added to the pretreated corn stover. The amount of cellulase was maintained at 15 FPU/g of dry matter. Multienzyme complex consists of arabinose, *β*-glucanase, cellulase, hemicellulase, pectinase, and xylanase, which has the ability to liberate bound materials and can degrade a variety of nonstarch polysaccharides. Hydrolysis was carried out for 72 hours at 50°C and 150 rpm as described by Selig et al. [42]. After hydrolysis, the samples were kept in boiling water for 10 min to inactivate the enzyme action. The supernatant was centrifuged at 13000 rpm for 15 min and then frozen twice before injecting into the HPLC to remove the impurities which contribute to the pressure increase in the HPLC system. Soluble sugars and byproducts were quantified using HPLC (Agilent Technologies, Santa Clara, CA; Bio-Rad Aminex 87H column Hercules, CA) with a mobile phase of 0.005 M H_2_SO_4_ at a flow rate of 0.6 mL/min at 65°C and a sample volume of 20 *μ*L as mentioned by Sluiter et al. [43].

Ground biomass was also subjected to enzymatic hydrolysis and analyzed as the control. The sugar concentration obtained from chromatogram was divided by dry weight of biomass taken for enzymatic hydrolysis in order to calculate the percentage of different sugars with respect to total biomass. Glucose and xylose are the major sugars present in the biomass as compared to arabinose. Instead of reporting arabinose separately, it was added with glucose and xylose and reported as combined sugar. Acetic acid and glycerol were the byproducts found in the pretreated samples and their concentration was reported in gL^−1^:

(1) $$\begin{array}{c} Y_{i}=\frac{S_{\text{ip}}}{S_{\text{ir}}}*100, \end{array}$$

(2) $$\begin{array}{c} Y_{c}=\frac{\sum S_{\text{ip}}}{\sum S_{\text{ir}}}*100, \end{array}$$

*Y*_*i*_: individual sugar recovery, %,
*Y*_*c*_: combined sugar recovery, %,
*S*_ip_: individual sugar obtained from pretreated samples through HPLC,
*S*_ir_: individual sugar from raw material.

### 2.4. Statistical Analysis

The moisture balanced big bluestem and prairie cord grass were extruded using a screw with 3 : 1 compression ratio at varying screw speeds of 100, 150, and 200 rpm and barrel temperatures of 100, 150, and 200°C, which resulted in nine treatment combinations (i.e., 3 × 3 = 9) per each biomass. The switchgrass was pretreated at three different screw speeds (100, 150, and 200 rpm) and the barrel temperatures of 50, 75, 100 and 150°C. Each treatment run was divided into two batches and the samples collected were considered as replicates. The collected data were analyzed with PROC GLM procedure to determine the main, interaction and treatment effect in SAS 9.1 (SAS Institute, Cary, NC) using a type I error (*α*) of 0.05.

## 3. Results and Discussion

### 3.1. Characterization of Different Biomasses

The switchgrass, big bluestem, and prairie cord grass composition such as glucose, xylose, arabinose, lignin, ash content on dry matter basis (%) were determined and given in Table 1. In general, glucose is referred as cellulose and xylose, arabinose, galactose, and mannose are combined together and termed as hemicellulose. The switchgrass used in this study had lower glucose content than the values reported by many researchers [21, 29–31, 33, 36, 37] and comparable with that of DOE's [32] and Jefferson et al. [6]. However, the xylose content was lower; arabinose and lignin content were higher than the values of studies listed in Table 1. The big bluestem composition was in agreement with the DOE's value except for ash content. The glucose, xylose, and arabinose content were less than the reported values [6, 38–40], whereas the lignin and ash content were higher than those studies. Prairie cord grass used in this study had a lower cellulose and hemicellulose compared to the values reported by Boe and Lee [41]. The lower glucose and higher lignin content offered more resistance for any pretreatment method. Lignin played a critical role not only in plant growth and development but also in biomass utilization; lignin restricted the degradation of structural polysaccharides through enzymatic hydrolysis. In general, the chemical composition of any biomass varies from place to place depending upon the agronomic practices, season, and varieties.

### 3.2. Effect of Screw Speed on Sugar Recovery from Different Biomasses

**Figure 1:**
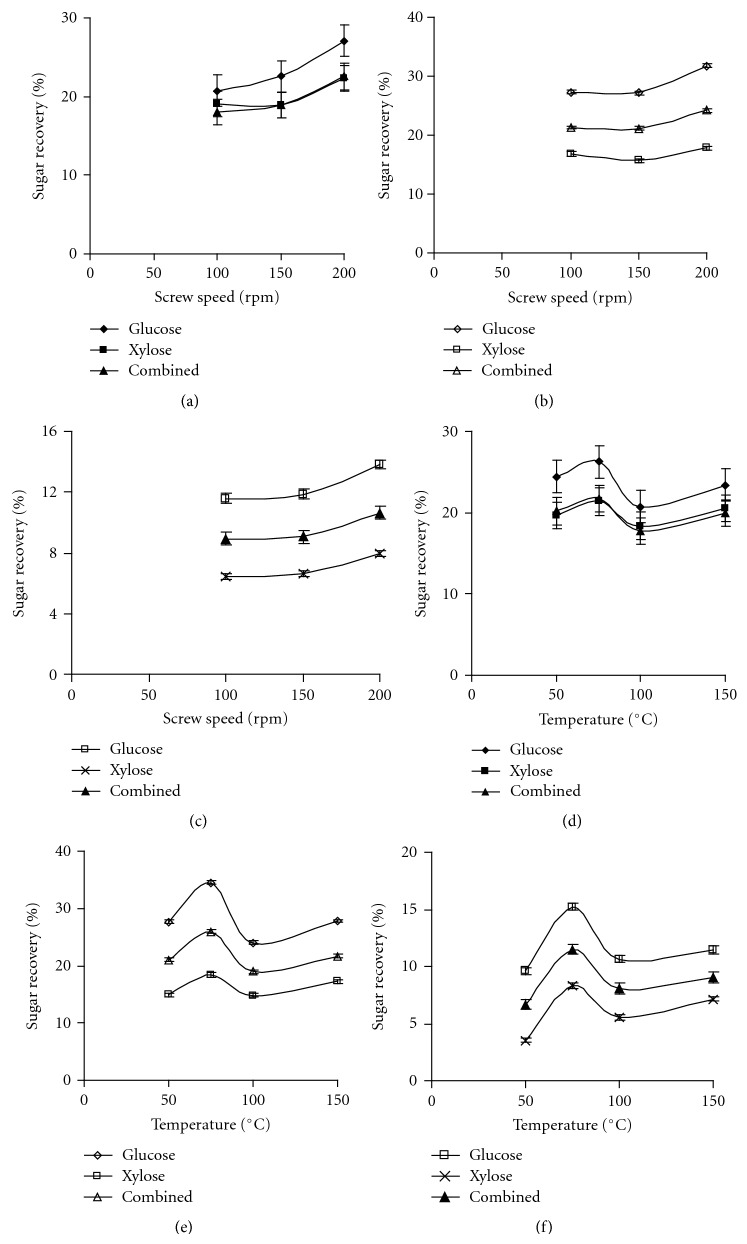
Effect of screw speed and barrel temperature on sugar recovery from switchgrass ((a, d)—1 : 1 cellulase and *β*-glucosidase, (b, e)—1 : 4 cellulase and *β*-glucosidase, and (c, f)—1 : 1 multienzyme and *β*-glucosidase).

The main effect analysis of screw speed on sugar recovery from switchgrass, big bluestem, and prairie cord grass is depicted in Figures 1(a)–1(c) through 3(a)–3(c) when cellulase, multienzyme, and *β*-glucosidase was used with different ratios during hydrolysis. In general, the screw speed had a positive influence on sugar recovery from switchgrass regardless of enzyme combinations and their ratios used during hydrolysis as seen from Figure 1. Muthukumarappan and Julson [14] reported sugar recovery increasing trend for switchgrass pretreated using a twin screw extruder while varying the screw speed from 200 to 400 rpm. Although the trend was same, they achieved lower sugar recovery than that of the present study. The difference might be due to type of extruder and pretreatment conditions, apart from chemical composition of switchgrass. In the present study, the change in sugar recovery was insignificant when the screw speed was increased from 100 to 150 rpm; however, further increase in screw speed had a significant increase only for glucose recovery when cellulase and *β*-glucosidase was used with 1 : 1 ratio (Figure 1(a)). When amount of *β*-glucosidase was increased by four times with cellulase amount kept constant, similar sugar recovery pattern was observed as that of 1 : 1 cellulase with *β*-glucosidase ratio (Figure 1(b)). Karunanithy et al. [15] reported a similar trend for corn stover pretreated in a single screw extruder. The glucose recovery increasing trend found in this study is in agreement with previous study of alkali-microwave pretreated switchgrass and bermudagrass [37]; however, the glucose recovery was less in the present study. The possible reason for higher amount of glucose recovered might be due to delignification of switchgrass by alkali. Statistical analysis showed that the increase in sugar recovery across the screw speed was not significant when cellulase and *β*-glucosidase was used at 1 : 4 ratio. The glucose recovery was comparable between cellulase with different *β*-glucosidase ratios, whereas xylose recovery differed among them. When multienzyme with *β*-glucosidase was used during hydrolysis, the screw speed showed significant difference on sugar recovery from switchgrass. The sugar recovery was less than 50% as compared to that of 1 : 1 ratio of cellulase with *β*-glucosidase (Figure 1(c)). When the screw was increased from 100 to 200 rpm, the glucose, xylose, and combined sugar recovery increased between 16–30, 6–23, and 14–25%, respectively, depending upon the enzyme combinations and their ratios. These results showed that the rate of shear development was more important than the mean residence time.

**Figure 2:**
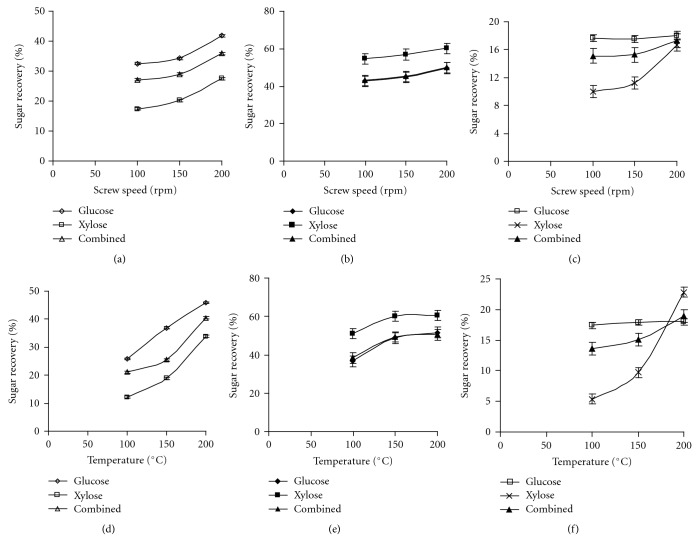
Effect of screw speed and barrel temperature on sugar recovery from big bluestem ((a, d)—1 : 1 cellulase and *β*-glucosidase, (b, e)—1 : 4 cellulase and *β*-glucosidase, and (c, f)—1 : 1 multienzyme and *β*-glucosidase).

As observed from Figure 2 the sugar recovery from big bluestem increased with an increase in screw speed across the enzyme combinations and ratios. The screw speed showed a significant difference on sugar recovery when 1 : 1 cellulase and *β*-glucosidase was used (Figure 2(a)). As the screw speed was increased from 100 to 200 rpm, the glucose, xylose, and combined sugar recovery also increased by 28.3, 57.4, and 32.2%, respectively. A similar result was reported by Muthukumarappan and Julson [14] for big bluestem pretreated using a twin screw extruder while varying the screw speed from 200 to 400 rpm. However, the sugar recovery obtained by these authors is less than that of the present study. The difference might be due to type of extruder and pretreatment conditions, apart from chemical composition of big bluestem. Higher sugar recovery was recorded when *β*-glucosidase amount was increased by four times while cellulase amount was maintained at same level. However, the increase in sugar recovery was not statistically different across the screw speeds as noticed in Figure 2(b). When multienzyme with *β*-glucosidase was used at a ratio of 1 : 1, the sugar recovery from big bluestem increased irrespective of the screw speeds. The increase in glucose and combined sugar recovery was negligible when 1 : 1 multienzyme with *β*-glucosidase was employed during hydrolysis (Figure 2(c)) whereas, 1 : 1 cellulase and *β*-glucosidase showed significant increase on sugar recovery. A significant improvement on xylose recovery was observed as the screw speed was increased from 150 to 200 rpm (Figure 2(c)). Multienzyme with *β*-glucosidase (1 : 1) resulted in a lower sugar recovery among the enzyme combinations and ratios studied, and it was almost less than 50% of cellulase with *β*-glucosidase (1 : 1). These results showed that higher screw speed is required to disturb the cell wall structure of the big bluestem.

**Figure 3:**
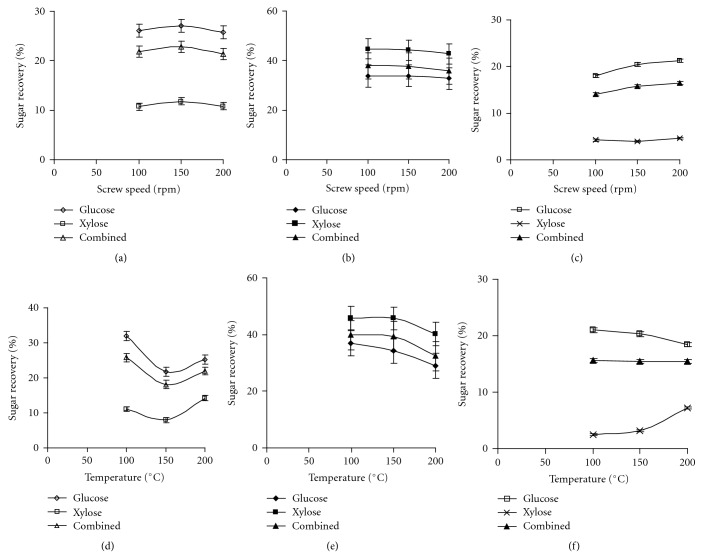
Effect of screw speed and barrel temperature on sugar recovery from prairie cord grass ((a, d)—1 : 1 cellulase and *β*-glucosidase, (b, e)—1 : 4 cellulase and *β*-glucosidase, and (c, f)—1 : 1 multienzyme and *β*-glucosidase).

The influence of screw speed on sugar recovery from prairie cord grass with different enzymes and ratios are depicted in Figure 3. In general, the sugar recovery decreased with an increase in screw speed when cellulase with *β*-glucosidase was used during hydrolysis, whereas sugar recovery increased with screw speed when multienzyme with *β*-glucosidase was used. The sugar recovery slightly increased as the screw speed was increased from 100 to 150 rpm; further increase in screw speed decreased the sugar recovery from prairie cord grass (Figure 3(a)). However, the change in sugar recovery was not statistically different across the screw speed. As the amount of *β*-glucosidase was increased by four times correspondingly the sugar recovery also increased. The increase in glucose recovery might be attributed to the higher amount of *β*-glucosidase, which effectively broke down the cellobiose to glucose. A remarkable increase in xylose recovery was noted when *β*-glucosidase amount was increased as evident from Figure 3(b). The statistical analysis showed that the decrease in sugar recovery across the screw speeds was not significant. These results showed that any screw speed could be selected for the pretreatment of prairie cord grass among the screw speeds studied. When cellulase was replaced with multienzyme, the sugar recovery dropped by 30% approximately. The sugar recovery decrease might be due to lower amount of cellulase present in the multienzyme. The screw speed showed a positive influence on glucose and combined sugar recovery from prairie cord grass when multienzyme with *β*-glucosidase was used at a ratio of 1 : 1, but screw speed had no influence on xylose recovery (Figure 3(c)).

### 3.3. Effect of Barrel Temperature on Sugar Recovery from Different Biomasses

Figures 1(d)–1(f) through 3(d)–3(f) represent the effect of barrel temperature on sugar recovery from different biomasses studied when cellulase, multienzyme was added with *β*-glucosidase during hydrolysis. The temperature had a significant effect on sugar recovery regardless of enzyme combinations and their ratios, except 1 : 1 cellulase to *β*-glucosidase ratio. Among the temperatures studied, the maximum sugar recovery was obtained at a temperature of 75°C. Statistical analysis of sugar recovery showed that the difference was not significant across the barrel temperatures when 1 : 1 cellulase to *β*-glucosidase ratio was employed during hydrolysis (Figure 1(d)). When *β*-glucosidase amount was increased by four times, a similar sugar recovery pattern was observed as compared to cellulase and *β*-glucosidase at a ratio of 1 : 1. The sugar recovery initially increased by 25% when temperature was increased from 50 to 75°C, and further increase in the barrel temperature reduced the sugar recovery (Figure 1(e)). The glucose recovery was higher with cellulase to *β*-glucosidase ratio of 1 : 4 compared to that of 1 : 1 ratio. The increase in glucose recovery might be due to the higher amount of *β*-glucosidase. When multienzyme was used instead of cellulase, the sugar recovery increased with an increase in temperature. The maximum sugar recovery was noticed at a barrel temperature of 75°C (Figure 1(f)) and it was similar to other enzyme combinations. Glucose, xylose, and combined sugar recovery increased by 19, 103, and 36%, respectively, when the barrel temperature was increased from 50 to 150°C while multienzyme and *β*-glucosidase was employed at ratio of 1 : 1. The sugar recovery was lowered by 45% when multienzyme and *β*-glucosidase was used during hydrolysis compared to cellulase and *β*-glucosidase with 1 : 1 ratio. These result showed that the cell wall disturbance was maximum at 75°C; further increase in temperature might have contributed for thermal softening of switchgrass. The present study results were contrary to the results reported for switchgrass pretreated in a twin screw extruder at a screw speed of 200 rpm while the temperature was increased from 25 to 100°C [14]. This might be due to the difference in type of extruder, pretreatment conditions, and chemical composition of switchgrass. 

The barrel temperature had a strong influence on the sugar recovery from big bluestem when cellulase and *β*-glucosidase was used at a ratio of 1 : 1 as evident from Figure 2(d). As the barrel temperature was increased from 100 to 200°C, the glucose, xylose, and combined sugar recovery correspondingly increased from 25.8, 12.2, 21.1 to 45.9, 33.9, and 40.5%, respectively. As the *β*-glucosidase amount was increased by four times while cellulase amount was kept constant, the sugar recovery increased with an increase in barrel temperature. Higher sugar recovery with higher *β*-glucosidase might be due to higher cellobiose broken into glucose. A remarkable increase in xylose recovery was noticed between cellulase with different ratios of *β*-glucosidase (Figures 2(d) and 2(e)). Statistical analysis showed a significant difference on sugar recovery between 100 and 150°C, further increase of barrel temperature resulted in negligible sugar recovery increase as seen from Figure 2(e). When multienzyme and *β*-glucosidase was used at a ratio of 1 : 1, the sugar recovery pattern was different than the cellulase and *β*-glucosidase combination. The barrel temperature had a significant influence on xylose recovery, whereas temperature had no influence on glucose recovery as noted from Figure 2(f). These results showed that irrespective of enzyme combination and ratios, higher barrel temperature was required for higher sugar recovery from big bluestem. In contrary to an increasing trend with barrel temperature, a decreasing trend was reported by Muthukumarappan and Julson [14] for big bluestem pretreated in a twin screw extruder when the barrel temperature increased from 25 to 100°C at a screw speed of 200 rpm. The glucose recovery from the present study was higher (37.1%) compared to Muthukumarappan and Julson's [14] result (27.2%) when big bluestem was pretreated at a barrel temperature of 100°C and screw speed of 200 rpm; the difference might be due to type of extruder and the chemical composition of big bluestem.

The barrel temperature negatively influenced the sugar recovery from prairie cord grass when cellulase and *β*-glucosidase was used for hydrolysis. The higher sugar recovery was obtained at lower barrel temperature as evident from Figure 3(d). When the temperature increased from 100 to 150°C, the glucose, xylose, and combined sugar recovery was decreased by 31.7, 27.9, and 29.8%, respectively, and further increase of barrel temperature resulted in small increase of sugar recovery. It was noted that xylose recovery was maximum at 200°C whereas glucose and combined sugar recovery was maximum at 100°C. In general, the sugar recovery from prairie cord grass increased when the amount of *β*-glucosidase was increased by four times (Figure 3(e)) which indicates the higher amount of *β*-glucosidase enhanced/facilitated in the conversion of cellobiose to glucose. Xylose recovery showed a remarkable increase of four times when compared to cellulase with *β*-glucosidase (1 : 1). The barrel temperature had a negative impact on sugar recovery from cord grass. However, the temperature effect was not significant on sugar recovery across the temperatures studied. When multienzyme and *β*-glucosidase was used at a ratio of 1 : 1, the sugar recovery decreased considerably when compared to cellulase and *β*-glucosidase at 1 : 1 ratio. A different sugar recovery pattern was observed with 1 : 1 multienzyme and *β*-glucosidase was employed during hydrolysis (Figure 3(f)). The barrel temperature of 100 and 150°C had no influence on glucose recovery, whereas the glucose recovery decreased at 200°C. Similar to glucose recovery, the lower temperature had no effect on xylose recovery, but the higher temperature showed a significant difference in xylose recovery. In contrary to cellulase enzyme combination, the barrel temperature effect was insignificant when multienzyme and *β*-glucosidase was employed during hydrolysis. These results showed that the lower barrel temperature would result in higher sugar recovery with cellulase and *β*-glucosidase combination.

### 3.4. Interaction and Treatment Effects on Sugar Recovery from Different Biomasses

The screw speed and barrel temperature interaction effect on sugar recovery from switchgrass, big bluestem, and prairie cord grass when different enzyme combinations and ratios were used during hydrolysis is given in Table 2. When cellulase or multienzyme with *β*-glucosidase was used at a ratio of 1 : 1, the temperature interaction with screw speed was different for switchgrass as evident from Table 2. Although temperature and screw speed as an independent variable had a significant effect on combined sugar from switchgrass, their interaction was not significant. A different interaction pattern was observed for big bluestem. When 1 : 1 cellulase and *β*-glucosidase combination was employed for hydrolysis, the temperature, screw speed, and their interaction had a significant impact on glucose, xylose, and combined sugar recovery. As the *β*-glucosidase amount was increased, only temperature had a significant influence on sugar recovery. The screw speed and temperature interaction was significant only for xylose recovery from big bluestem when multienzyme combination was used during hydrolysis. Although temperature had a significant influence on combined sugar recovery for big bluestem when it interacted with screw speed, the influence was negligible. The barrel temperature and screw speed interaction was not significant on sugar recovery from prairie cord grass when cellulase enzyme combination was employed during hydrolysis. When multienzyme combination was used for hydrolysis, the interaction effect was significant for glucose and combined sugar recovery from prairie cord grass. Although temperature as an independent variable had no significant effect on combined sugar recovery from cord grass, when it interacted with screw speed the effect turned to be significant.

Statistical analyses across the treatment combinations are presented in Tables 3 through 5 for different enzymes and ratios employed during hydrolysis of different biomasses. The temperature, screw speed, and their interaction had influence on sugar recovery from switchgrass as confirmed from statistical analysis. In general, the barrel temperature of 75°C showed higher sugar recovery from switchgrass (Table 3) across the screw speeds regardless of enzyme combinations and ratios. The maximum glucose, xylose, and combined sugar recovery of 38.7, 18.2, and 28.2%, respectively, was obtained at a screw speed of 200 rpm and barrel temperature of 75°C when cellulase and *β*-glucosidase was employed at a ratio of 1 : 4. Various pretreatments employed on switchgrass, enzyme dose, glucose, xylose, and total sugar yields are listed in Table 6. The glucose recovery from switchgrass was less than that of the ammonia-water pretreatment results (55.2 and 43.7%) for two switchgrass varieties [30]; 93% glucose from ammonia fiber expansion [31]; 91.4% glucose from dilute acid pretreatment [44] and 73–86% glucose from another dilute-acid pretreatment of switchgrass [45]. A glucose recovery of 68 and 87% for switchgrass pretreated in a combination of alkali and RF heating, alkali and microwave heating was reported by Hu and Wen [34] and Hu et al. [35], respectively, depending upon the alkali concentration and pretreatment conditions. It is a well-known fact that alkali removes the lignin and acid solubilizes the hemicellulose thereby increasing the accessibility for the enzymes resulting in higher glucose recovery.

As inferred from Table 4 that the higher screw speed combined with higher temperature resulted in higher glucose, xylose, and combined sugar recovery from big bluestem. However, the maximum glucose (57.5%), xylose (66.2%), and combined sugar recovery (57.6%) from big bluestem were achieved at a barrel temperature of 150°C and screw speed of 200 rpm when cellulase to *β*-glucosidase ratio was maintained at 1 : 4. This result was higher than the glucose availability of 27.2 and 26.8% reported for 20% moisture content big bluestem extruded in a twin screw extruder at a barrel temperature of 100°C with 200 and 400 rpm, respectively [14]. The difference might be due to the type of extruder and pretreatment conditions employed. No regular trend was observed on sugar recovery from prairie cord grass (Table 5). However, the higher glucose recovery was noticed at lower barrel temperature. A maximum glucose, xylose, and combined sugar recovery of 41.1, 49.2, and 44.6%, respectively, were recorded for prairie cord grass pretreated at a barrel temperature of 100°C and screw speed of 150 rpm when cellulase and *β*-glucosidase was employed at a ratio of 1 : 4.

### 3.5. Comparison of Sugar Recovery from Switchgrass, Big Bluestem, and Prairie Cord Grass

Switchgrass and big bluestem showed a similar pattern (increasing) of sugar recovery with an increase in screw speed, whereas the sugar recovery pattern was different for prairie cord grass. The sugar recovery from switchgrass showed an increasing trend with screw speed and it was similar to corn stover [12]. Switchgrass and big bluestem exhibited an increasing sugar recovery trend with temperature increase from 100 to 150°C. However, the increase was higher for big bluestem as compared to switchgrass. This observation was in agreement with Karunanithy et al. [12] reported for corn stover pretreated in a single screw extruder. Prairie cord grass showed decreasing sugar recovery trend with an increase in temperature. The glucose, xylose, and combined sugar recovery differed among switchgrass, big bluestem, and prairie cord grass, which might be due the difference in their chemical composition and inherent nature of biomasses. The lowest and highest sugar recovery was recorded for switchgrass (38.7, 28.2%) and big bluestem (57.5, 57.6%), respectively. The lowest sugar recovery from switchgrass might be attributed to the highest lignin content among the biomasses studied. In general, an increase in glucose recovery with *β*-glucosidase from the biomasses studied was in agreement with microwave-alkali pretreated switchgrass and bermudagrass reported by Keshwani [37]. 

### 3.6. Byproducts Formation

Glycerol and acetic acid were the byproducts found in a few pretreated switchgrass, big bluestem, and prairie cord grass samples. The concentration of glycerol and acetic acid were in the range of 0.02–0.05 and 0.02–0.04 gL^−1^, respectively, recorded for switchgrass at a barrel temperature of 50°C with screw speeds of 100 and 150 rpm. The maximum concentration of glycerol and acetic acid was 0.03 and 0.04 gL^−1^, respectively, and was recorded for big bluestem at a screw speed of 150 rpm and barrel temperature of 100°C. The concentration of glycerol and acetic acid was similar to switchgrass but occurred at higher temperature and screw speed. In contrary to other extrusion pretreatments of corn stover, miscanthus and Douglas fir wood, acetic acid and glycerol were found in this study. Interestingly no furfural and HMF were found in any of the pretreatment conditions studied, which was in agreement with other extrusion pretreatments performed on different biomasses [11, 13, 16].

## 4. Extruder Energy Calculation

It consists of two components, namely, drive and heaters. The extruder has a power source of 7.5 hp (5595 W). There are 4 heaters per extruder zone at 250 W each, so Zone 1 = 1000 W and Zone 2 = 1000 W. Thus, the total 20 : 1 *l*/*d* extruder wattage is 2000 W. The total rated power to run this extruder for an hour is 7595 Watt-hour. The ideal/no load drive and heater power consumption are 1.4 and 0.8 amps, respectively. The actual drive power consumption is 3.5, 4.4, and 4.1 amps and heater power consumption is 1.6, 2.4, and 2 amps, respectively, for big bluestem, prairie cord grass, and switchgrass. For three phase alternate current, *W* = $\sqrt{3VI}$, where *V* is voltage (240 V) and *I* is current in amps. The total energy consumption for extrusion pretreatment of big bluestem, prairie cord grass, and switchgrass is 2120, 2826, and 2515 W, respectively, which is 27.9, 37.2, and 33.1% of the rated drive and heater power.

This extruder can pretreat 2-3 kgh^−1^ depending on the types of feedstock. Amount of ethanol production can be calculated based on the availability of glucose in the raw feedstock and glucose recovered through extrusion pretreatment. Assumping a thumb rule is 50% of the glucose will be converted into ethanol during fermentation with an efficiency of 90%. Ethanol density and its energy content are 0.79 g/mL and 21.1 MJL^−1^, respectively (1 MJ = 278 Watt-hour). For example, glucose recovery of 57.5, 38.7, and 41.1% recorded for big bluestem, switchgrass, and prairie cord grass, respectively, would result in 1349, 909, and 809 Watt-hour. Researchers across the world are working on utilizing hemicellulose fraction; once the technology is developed, one can expect an additional energy of 60% that would narrow down the difference between input and output. However, extruder used in this study is a lab-scale one; the efficiency would improve as a result of scale-up process, and thereby one can expect net positive energy balance. In conclusion, technology development for hemicellulose utilization and scale-up process would be need of the hour.

## 5. Conclusion

This experiment was conducted to understand the influence of screw speed and barrel temperature on sugar recovery from switchgrass, big bluestem, and prairie cord grass. When different enzyme combinations and ratios were employed during hydrolysis, it was confirmed that screw speed, barrel temperature and their combinations significantly influenced the sugar recovery. Based on the highest glucose, xylose, and combined sugar recovery, switchgrass could be pretreated at a screw speed of 200 rpm with barrel temperature of 75°C with cellulase and *β*-glucosidase at a ratio of 1 : 4. The big bluestem could be pretreated at a screw speed of 200 rpm with 150°C, and prairie cord grass could be pretreated at a screw speed of 150 rpm and 100°C while cellulase and *β*-glucosidase was employed at a ratio of 1 : 4. Pretreatment of these biomasses used 28–37% of the rated extruder power. Further studies will be conducted to improve the sugar recoveries from this biomass.

## Figures and Tables

**Table 1:** Chemical composition of switchgrass, big bluestem, and prairie cord grass on dry matter basis (%).

Biomass	Glucose	Xylose	Arabinose	Lignin	Ash	Reference
Switchgrass	25.5 ± 5.8	17.4 ± 2.1	4.9 ± 1.2	24.7 ± 2.1	2.9 ± 0.06	Present study
Switchgrass	38.0	22.0^*^		22.0	6.0	[21]
Switchgrass	31.3	20.6	3.1	21.4	7.1	[29]
Switchgrass	41.4	20.9^*^		17.3		[30]
Switchgrass	31.8	30.6^*^			8.5	[6]
Switchgrass	34.1	22.1	3.1^+^			[31]
Switchgrass	31.0–35.4	20.4–24.0	2.7–3.3	17.4–20.8	4.5–7.5	[32]
Switchgrass	34.2 ± 2.1	22.8 ± 1.0	3.1 ± 0.5	19.1 ± 1.7	5.9 ± 1.0	[33]
Switchgrass	33.6 ± 1.0	19.3 ± 0.6		21.4 ± 0.8	3.9 ± 0.3	[34, 35]
Switchgrass	42.0	31.0^*^		22.0	7.0	[36]
Switchgrass	31.3	18.4	1.9	22.5		[37]

Big bluestem	21.1 ± 7.2	8.82 ± 1.4	2.45 ± 0.2	21.1 ± 0.6	11.2 ± 0.05	Present study
Big bluestem	42.5	25.9	4.5	21.0		[38]
Big bluestem	34.7	29.2^*^			8.0	[6]
Big bluestem	37.0	28.0^*^		18.0	6.0	[39, 40]
Big bluestem	29.0–37.2	15.7–22.6	2.4–3.6	17.1–23.8	2.8–5.1	[32]

Prairie cord grass	33.1 ± 0.4	13.5 ± 2.0	1.6 ± 0.6	21.0 ± 0.5	5.6 ± 0.04	Present study
Prairie cord grass	41	33^*^			6.0	[41]

^*^Hemicellulose: sum of xylose, arabinose, galactose, and mannose.

^+^Combination of arabinose and galactose.

**Table 2:** Interaction effects (*P* value) of screw speed and barrel temperature on sugar recovery from different biomasses.

Source	Switchgrass			Big bluestem			Prairie cord grass		
	Glucose	Xylose	Combined	Glucose	Xylose	Combined	Glucose	Xylose	Combined
1 Cellulase : 1 *β*-glucosidase									

Temp	0.2115	0.4627	0.2793	<0.0001	<0.0001	<0.0001	0.0011	0.0008	0.0040
Screw speed	0.1277	0.3122	0.1752	<0.0001	<0.0001	<0.0001	0.7399	0.5537	0.6705
Temp∗screw speed	0.4888	0.4940	0.5078	<0.0001	0.0002	<0.0001	0.7055	0.2894	0.7271

1 Cellulase : 4 *β*-glucosidase									

Temp	<0.0001	<0.0001	<0.0001	0.0110	0.0468	0.0234	0.4655	0.5518	0.5633
Screw speed	<0.0001	0.0046	<0.0001	0.2526	0.3593	0.2526	0.9793	0.9417	0.9522
Temp∗screw speed	0.0043	0.0102	0.0765	0.3452	0.5526	0.3654	0.5521	0.6598	0.6163

1 Multienzyme : 1 *β*-glucosidase									

Temp	<0.0001	<0.0001	0.0011	0.6948	<0.0001	0.0137	0.0032	<0.0001	0.4914
Screw speed	0.0012	0.0008	0.0412	0.7554	0.0006	0.3020	0.0005	0.1499	<0.0001
Temp∗screw speed	0.0002	0.0109	0.1987	0.7897	0.0322	0.7827	0.0017	0.4073	<0.0001

**Table 3:** Effect of screw speed and temperature on sugar recovery from switchgrass.

Screw speed, rpm	Temperature, °C											
	50	75	100	150	50	75	100	150	50	75	100	150
	1 cellulase : 1 *β*-glucosidase				1 cellulase : 4 *β*-glucosidase				1 multienzyme : 1 *β*-glucosidase			
Glucose												

100	25.1^a^	21.7^a^	18.6^a^	21.9^a^	30.6^cd^	31.4^c^	22.8^h^	27.7^f^	7.6^e^	12.5^bc^	11.0^cd^	11.2^cd^
150	23.7^a^	29.7^a^	18.5^a^	19.6^a^	24.8^g^	33.2^b^	21.9^h^	26.5^fg^	11.3^cd^	13.3^b^	10.5^d^	11.7^b–d^
200	NA	27.3^a^	25.1^a^	28.8^a^	NA	38.7^a^	27.3^f^	29.2^de^	NA	19.7^a^	10.4^d^	11.4^cd^

Xylose												

100	19.3^a^	20.3^a^	15.9^a^	21.0^a^	16.7^bc^	19.9^a^	13.3^d^	17.2^bc^	2.5^d^	8.2^a^	4.2^c^	6.9^b^
150	19.9^a^	22.9^a^	17.2^a^	16.6^a^	13.2^d^	17.1^bc^	13.8^d^	16.3^c^	4.5^c^	8.4^a^	5.0^c^	6.5^b^
200	NA	20.9^a^	22.1^a^	24.0^a^	NA	18.2^ab^	17.2^bc^	18.2^ab^	NA	8.4	7.5^ab^	8.1^a^

Combined												

100	20.4^a^	19.0^a^	15.7^a^	19.4^a^	23.2^bc^	24.7^b^	17.4^e^	21.7^cd^	5.3^d^	10.2^bc^	7.7^c^	8.9^bc^
150	19.9^a^	24.2^a^	16.1^a^	16.5^a^	18.9^e^	24.9^b^	17.9^e^	20.7^d^	8.0^bc^	10.4^b^	7.8^c^	8.9^bc^
200	NA	22.1^a^	21.5^a^	24.1^a^	NA	28.2^a^	21.7^cd^	22.7^c^	NA	14.0^a^	8.7^bc^	9.3^bc^

Control: 19.4% glucose, 16.4% xylose, and 20.6% combined sugar (with 1 : 4 cellulase to *β*-glucosidase). Superscripts with same letter within column are not significantly different for each sugar under each enzyme combination. NA—not applicable.

**Table 4:** Effect of screw speed and temperature on sugar recovery from big bluestem.

Screw speed, rpm	Temperature, °C								
	100	150	200	100	150	200	100	150	200
	1 cellulase : 1 *β*-glucosidase			1 cellulase : 4 *β*-glucosidase			1 multienzyme : 1 *β*-glucosidase		
Glucose									

100	17.8^e^	33.0^c^	46.9^a^	31.2^c^	43.3^a–c^	53.3^ab^	17.5^a^	17.4^a^	18.2^a^
150	25.7^d^	34.6^c^	42.6^b^	41.7^a–c^	45.3^a–c^	47.2^a–c^	17.6^a^	17.7^a^	17.3^a^
200	33.9^c^	43.2^b^	48.2^a^	37.1^bc^	57.5^a^	54.3^ab^	17.1^a^	18.6^a^	18.5^a^

Xylose									

100	9.6^e^	10.7^e^	32.0^b^	46.7^b^	56.2^ab^	60.9^ab^	3.8^e^	4.0^e^	22.2^b^
150	10.3^e^	18.0^d^	32.2^b^	55.2^ab^	57.8^ab^	57.5^ab^	5.4^de^	8.9^d^	19.4^bc^
200	16.8^d^	28.1^c^	37.4^a^	50.9^ab^	66.2^a^	63.3^a^	6.8^de^	16.3^c^	26.8^a^

Combined sugar									

100	15.0^e^	25.6^c^	40.8^a^	33.1^c^	44.6^a–c^	51.4^ab^	13.3^b^	13.2^b^	18.9^a^
150	20.4^d^	13.8^e^	37.8^b^	43.4^a–c^	45.7^a–c^	46.5^a–c^	13.6^b^	14.7^ab^	17.5^a^
200	27.8^c^	36.9^b^	42.9^a^	38.7^a–c^	57.6^a^	53.2^ab^	13.9^b^	17.5^a^	20.4^a^

Control: 20.3% glucose, 34.1% xylose, and 22.5% combined sugar (with 1 : 4 cellulase to *β*-glucosidase). Superscripts with same letter within column are not significantly different for each sugar under each enzyme combination.

**Table 5:** Effect of screw speed and temperature on sugar recovery from prairie cord grass.

Screw speed, rpm	Temperature, °C								
	100	150	200	100	150	200	100	150	200
	1 cellulase : 1 *β*-glucosidase			1 cellulase : 4 *β*-glucosidase			1 multienzyme : 1 *β*-glucosidase		
Glucose									

100	31.7^ab^	22.2^cd^	24.4^b–d^	32.4^a^	32.2^a^	36.7^a^	16.3^f^	20.2^cd^	17.5^c–e^
150	34.5^a^	20.9^d^	25.9^b–d^	41.1^a^	39.2^a^	21.3^a^	22.8^ab^	19.5^c–e^	19.1^c–e^
200	29.6^a–c^	22.2^cd^	25.3^b–d^	37.3^a^	31.7^a^	29.1^a^	23.9^a^	21.1^bc^	18.8^de^

Xylose									

100	12.1^ab^	7.9^b^	12.1^ab^	43.5^a^	43.9^a^	46.4^a^	2.2^c^	3.0^bc^	7.4^a^
150	11.9^ab^	7.8^b^	15.6^a^	49.2^a^	50.0^a^	33.6^a^	2.7^bc^	2.7^bc^	6.6^a^
200	9.3^b^	8.3^b^	14.9^a^	44.8^a^	43.1^a^	40.3^a^	2.5^bc^	3.8^b^	7.7^a^

Combined sugar									

100	25.6^ab^	18.8^bc^	21.0^a–c^	34.7^a^	37.8^a^	41.2^a^	12.3^e^	15.3^cd^	14.8^d^
150	28.0^a^	17.3^c^	23.1^a–c^	44.6^a^	44.5^a^	24.0^a^	17.0^a^	14.7^d^	15.7^bc^
200	23.9^a–c^	18.3^c^	21.8^a–c^	39.9^a^	35.7^a^	31.8^a^	17.67^a^	16.2^b^	15.8^bc^

Control: 20.9% glucose, 33.8% xylose, and 23.8% combined sugar (with 1 : 4 cellulase to *β*-glucosidase). Superscripts with same letter within column are not significantly different for each sugar under each enzyme combination.

**Table 6:** Various methods used for pretreatment of switchgrass along with enzyme dose and reported results.

Pretreatment	Pretreatment condition	Cellulase, FPU/g DM	*β*-glucosidase CBU/g DM	Glucose %	Xylose %	Total %	Reference
Lime		5	28.4	60	72	64	
	0.1 Ca(OH)_2_ g^−1^, 120°C, 2 h, 9 mL water/g	10	28.4	68	80	70	
		25	28.4	76	90	80	[21]
		50, 75, 100	28.4	80	>90	84	

Lime	0.1 Ca(OH)_2_ g^−1^, 121°C, 15 min	38	66.5			75.8	[44]
AFEX	1 : 1 ammonia to switchgrass with 80% moisture, 100°C, 5 min (hydrolysis 7 days)	15^*^	40^*^	93	70		[31]
Alkali-microwave	0.1 g NaOH/g, 190°C, 2 h, solid loading 50 gL^−1^	12	21	87.3	95.4	90.2	[34]
Alkali-RF	0.2–0.25 g NaOH/g, 90°C, 20% solid loading	12	21	67.8	96.8	78.5	[35]

Aqueous ammonia soaking		26	NR			44	
	5 mL aqueous ammonium hydroxide g^−1^, 5 days (hydrolysis 4 days)	38.5	NR			68	
		77	NR			60	
		26	NR			60	
	10 mL aqueous ammonium hydroxide g^−1^, 10 days (hydrolysis 4 days)	38.5	NR			72	[36]
		77	NR			65	

Alkali-microwave		2.5	70	31		44.7	
		5	70	53.5		55.5	
		10	70	67.6		75.0	
	2% NaOH, 250 W, 10 min	15	70	81.7		80.5	[37]
		20, 40	70	82.0		80.0	
		15	10	67.6			
		15	20	82.0	63	80	
		15	40, 70	80.0			

DM—dry matter, ^*^per gram of cellulose, NR—not reported.
